# Validation and implementation of ambulatory obstructive sleep apnea polygraphy screening combined with wearable semi-continuous heart rhythm monitoring in patients with atrial fibrillation: a validation and a pilot study

**DOI:** 10.3389/fcvm.2026.1873057

**Published:** 2026-06-26

**Authors:** Anouk Delaet, Rana Önder, Paulien Vermunicht, Lieselotte Knaepen, Michiel Delesie, Johan Verbraecken, Karolien Weytjens, Paul Dendale, Johan Vijgen, Hein Heidbuchel, Lien Desteghe

**Affiliations:** 1 Faculty of Medicine and Life Sciences, Hasselt University, Hasselt, Belgium; 2 Department of Cardiology, Jessa Hospital, Hasselt, Belgium; 3 Department of Cardiology, Antwerp University Hospital, Antwerp, Belgium; 4 Research Group Cardiovascular Diseases, University of Antwerp, Antwerp, Belgium; 5 AZ Sint-Lucas Ghent, Ghent, Belgium; 6 Multidisciplinary Sleep Disorders Center, Antwerp University Hospital and University of Antwerp, Edegem, Belgium; 7 Sleep Center Hasselt, Jessa Hospital, Hasselt, Belgium; 8 Center for Research and Innovation in Care (CRIC), Department of Nursing and Midwifery Sciences, University of Antwerp, Antwerp, Belgium

**Keywords:** atrial fibrillation, cardiac monitoring, mHealth, obstructive sleep apnea, polygraphy, screening

## Abstract

**Background:**

Obstructive sleep apnea (OSA) is a prevalent yet underdiagnosed risk factor in atrial fibrillation (AF) patients and may influence AF burden. Systematic OSA screening and long-term non-invasive heart rhythm monitoring to follow up on AF remain challenging in clinical practice. This study aimed to validate the NOX-T3s polygraphy (PG) device for OSA screening, and to evaluate the feasibility of a structured OSA screening pathway combined with semi-continuous heart rhythm monitoring for mapping AF recurrences and burden.

**Methods:**

In the NOX-T3s validation study, NOX-T3s performance was evaluated in 30 AF patients undergoing PSG, with simultaneous and consecutive home NOX-T3s recordings. In the NOXFib-AF pilot study, the NOX-T3s was used for ambulatory OSA screening in another 30 AF patients, followed by 31 days of semi-continuous rhythm monitoring (every 9 min) via a smartwatch linked to the FibriCheck algorithm and twice-daily spot-checks via the FibriCheck smartphone application. Presence of AF was indicated as ≥1 AF measurement, with episodes defined as consecutive AF measurements terminated by a sinus rhythm measurement. Patients who screened OSA-positive were referred for PSG. Patient comfort with the devices was assessed via questionnaires.

**Results:**

NOX-T3s demonstrated good diagnostic performance for detecting moderate-to-severe OSA (AUC 0.83 simultaneous; 0.80 home), with Apnea-Hypopnea Index (AHI) values comparable to PSG (simultaneous recording *Δ*0.5 (IQR:−4.2–3.3); *p* *=* 0.942; separate-night recordings *Δ*1.5 (IQR:−3.7–7.4); *p* *=* 0.306). An optimized AHI cut-off of 11.1 events/h improved NOX-T3s accuracy (0.85), with high sensitivity (92.9%) and specificity (75.0%). OSA screening was successful in the NOXFib-AF study in 97.1% of patients, identifying moderate-to-severe OSA in 79.3%. 91.3% underwent PSG, confirming OSA in 61.9%. Semi-continuous smartwatch-based rhythm monitoring was successful in 96.6%, 61.0% ± 11.5 was high-quality data, and there was high compliance and motivation. AF was detected in 64.3%, and the smartwatch detected 6.7 times more AF episodes compared to spot-check alone. Comfort was high for both NOX-T3s (32 out of 40 (IQR: 29–35) and rhythm monitoring [34 out of 40 (IQR: 29–37)].

**Conclusions:**

NOX-T3s with an AHI cut-off ≥11.1 events/h is a reliable home screening tool for moderate-to-severe OSA. NOX-T3s-based OSA screening combined with semi-continuous FibriCheck-based rhythm monitoring has proven feasible and acceptable in AF patients.

## 1 Introduction

Obstructive sleep apnea (OSA) is an important yet underrecognized modifiable risk factor in patients with atrial fibrillation (AF) (1, 2). The expected prevalence of clinically relevant OSA, defined as moderate-to-severe OSA with an Apnea-Hypopnea Index (AHI) of ≥15 events/hour, is estimated to be 42.1% to 56.1% in patients with AF (3, 4). Of those, only 12.3% receiving a formal diagnosis in current practice (3, 4). This is worrisome, as patients with untreated OSA may respond poorly to AF treatment and have an increased risk of AF recurrence after rhythm-restoring procedures (5). Retrospective studies suggest that OSA treatment with continuous positive airway pressure (CPAP) reduces AF recurrence and progression. However, robust prospective evidence remains scarce and evaluating the impact of OSA and its treatment on AF outcomes such as recurrence and burden, requires long-term rhythm monitoring (6–11).

This day, long-term heart rhythm monitoring remains challenging in clinical practice (11). Traditional rhythm monitoring methods are often limited by short monitoring duration [e.g., Holter or electrocardiogram (ECG) patch monitor], high cost, or patient invasiveness (e.g., Implantable Loop Recorder). These limitations result in a substantial risk of underdetection of paroxysmal and often asymptomatic AF episodes in clinical practice (12). To address these shortcomings, heart rhythm monitoring using mobile Health technologies has emerged as an innovative, reliable, scalable, cost-effective, and non-invasive approach for prolonged rhythm monitoring in patients with AF, with accelerated adoption observed during the COVID-19 pandemic (13–15). These technologies include smartphone applications and wrist-worn devices that monitor the heart rhythm via photoplethysmography (PPG) or ECG (16). One of those technologies is FibriCheck®, a medical analysis platform that utilizes an end-to-end algorithm to detect AF based on PPG signals recorded on smartphones and smartwatches (17). Via the smartphone application, patients perform on-demand “spot-checks” by placing their finger on the camera of the smartphone and using the smartphone's flashlight to monitor heart rhythm from peripheral pulsatile blood volume changes. This approach has demonstrated high diagnostic performance, with a sensitivity of 93.4% and specificity of 99.4% (17). However, its reliance on patient-initiated measurement reduces the granularity of rhythm monitoring. This limitation is addressed by integrating the validated PPG algorithm into smartwatch technology, enabling (semi-) continuous non-intrusive rhythm monitoring throughout daily life while maintaining high diagnostic accuracy (sensitivity of 93.4% and specificity of 98.4%) (18).

According to the AF-CARE framework of the 2024 European Society of Cardiology (ESC) Guidelines on the management of AF, detecting and managing different risk factors that lead to AF, including OSA, are the primary focus for improving prognosis (15). However, they do not specify how to test for OSA and which tools are ideally suited (15). Polysomnography (PSG), the gold standard for OSA diagnosis, is too resource-intensive to serve as a screening tool in a large AF population (19). Therefore, there is a need for accurate and easy-to-use tools to test for OSA that can be integrated into daily ambulatory AF care. Cardiorespiratory polygraphy (PG) devices (level 3) are increasingly popular as screening tools and are recommended over screening questionnaires (1, 3, 19–21). These simplified portable devices can be used at home to record cardio-respiratory variables, which can facilitate the detection of OSA in a broader AF population and enabling earlier referral to sleep clinics for PSG confirmation and initiation of OSA treatment. This is essential, as in Belgium and other countries, treatment initiation still requires a PSG evaluation.

In a prior study of our research group (CarpOSAF study), the performance of three different PG devices with automated analyses (ApneaLink Air, SOMNOtouch RESP, and SpiderSAS), was validated in 100 patients with AF to determine the most reliable and patient-friendliest screening tool for clinically relevant OSA (22). This study found that home-worn PGs with an automated AHI algorithm and appropriate PG-specific cut-off values can be used as a reliable OSA screening tool in patients with AF, with the ApneaLink Air being the most accurate and user-friendly (22). Unfortunately, after the study, the AirView software, together with the ApneaLink Air, was no longer supported in Belgium. A similar PG device, the NOX-T3s, launched in 2020, offers comparable features. Although the NOX-T3s with an automated scoring algorithm have been validated in the general population, evidence in patients with AF who are often excluded from validation studies due to cardiorespiratory comorbidities is lacking (14, 23–25).

In a first study (i.e., NOX-validation study), we evaluated the diagnostic performance and accuracy of the NOX-T3s PG device as a reliable screening tool for clinically relevant OSA in patients with AF. In a second, separate follow-up NOXFib-AF pilot study, we examined the feasibility and user-friendliness of implementing the NOX-T3s device for ambulatory OSA screening combined with long-term semi-continuous smartwatch-based heart rhythm monitoring in routine AF care. The insights from these pilot studies serve as a foundation for the design and implementation of the currently ongoing ’Structured testing and treatment of obstructive sleep apnea in patients with atrial fibrillation’ (STAROSA) study (NCT06263608), which assesses the effect of OSA screening and treatment on AF burden in patients with AF and clinically relevant OSA.

## 2 Methods

### 2.1 Study design and study population

The NOX-T3s validation study (Belgian Registration number: B300201835708) and the NOXFib-AF pilot study (B3002023000024) are both multicenter, prospective, interventional cohort studies performed at the Antwerp University Hospital (UZA; Antwerp, Belgium) and Jessa Hospital (Jessa; Hasselt, Belgium). Both studies obtained ethical approval from the Ethics Committees of the participating centres and complied with the Declaration of Helsinki, and all participants provided written informed consent prior to enrolment.

The NOX-T3s validation study is a follow-up trial on the previously published CarpOSAF study by our research group, following the same methodology to validate another PG device, i.e., the NOX-T3s (Nox Medical, Reykjavik, Iceland) (22). For the validation study, 30 patients (15 participants per center) diagnosed with AF or atrial flutter with proven diagnosis on an ECG who were planned for a PSG examination for an evaluation of sleep-disordered breathing were recruited before the planned PSG at the sleep clinic of UZA and Jessa Hospital from January 2022 until October 2022. Exclusion criteria consisted of (1) not being able to speak and read Dutch, (2) age <18 years, (3) physical/cognitive impairment, and (4) participation in other clinical studies.

The second study, the NOXFib-AF pilot study, was designed to evaluate the feasibility and user-friendliness of implementing the NOX-T3s device for ambulatory OSA screening combined with long-term semi-continuous heart rhythm monitoring in routine AF care. For this study, 30 patients (18 participants from Jessa and 12 participants from UZA) with symptomatic [modified European Heart Rhythm Association score (mEHRA) ≥ 2a] paroxysmal or persistent AF and a proven AF diagnosis on an ECG were recruited from the cardiology outpatient clinic between May 2023 and August 2023. Patients were eligible if they owned a smartphone compatible with the Fitbit® and FibriCheck® applications. Exclusion criteria were (1) diagnosis with *de novo* AF, or permanent AF, or only atrial flutter, (2) prior PSG evaluation (< 5 year and no big differences in BMI or OSA symptoms), (3) CPAP treatment, (4) cognitive impairment, (5) inability to speak or fully understand Dutch, and (6) pacemaker-dependent heart rhythm. All demographic data (age, sex, BMI) and AF related parameters (type of AF, time since AF diagnosis, mEHRA symptom classification, CHA_2_DS_2_-VASc score, HAS-BLED score, AF treatment) were collected from the patients’ medical records. Physical parameters such as patient's neck and waist circumference were also assessed. The type of smartphone and operating system were recorded during the study visit at which the FibriCheck application was installed on the patient's smartphone.

### 2.2 Study procedure

#### 2.2.1 NOX-T3s validation study

**Figure 1:**
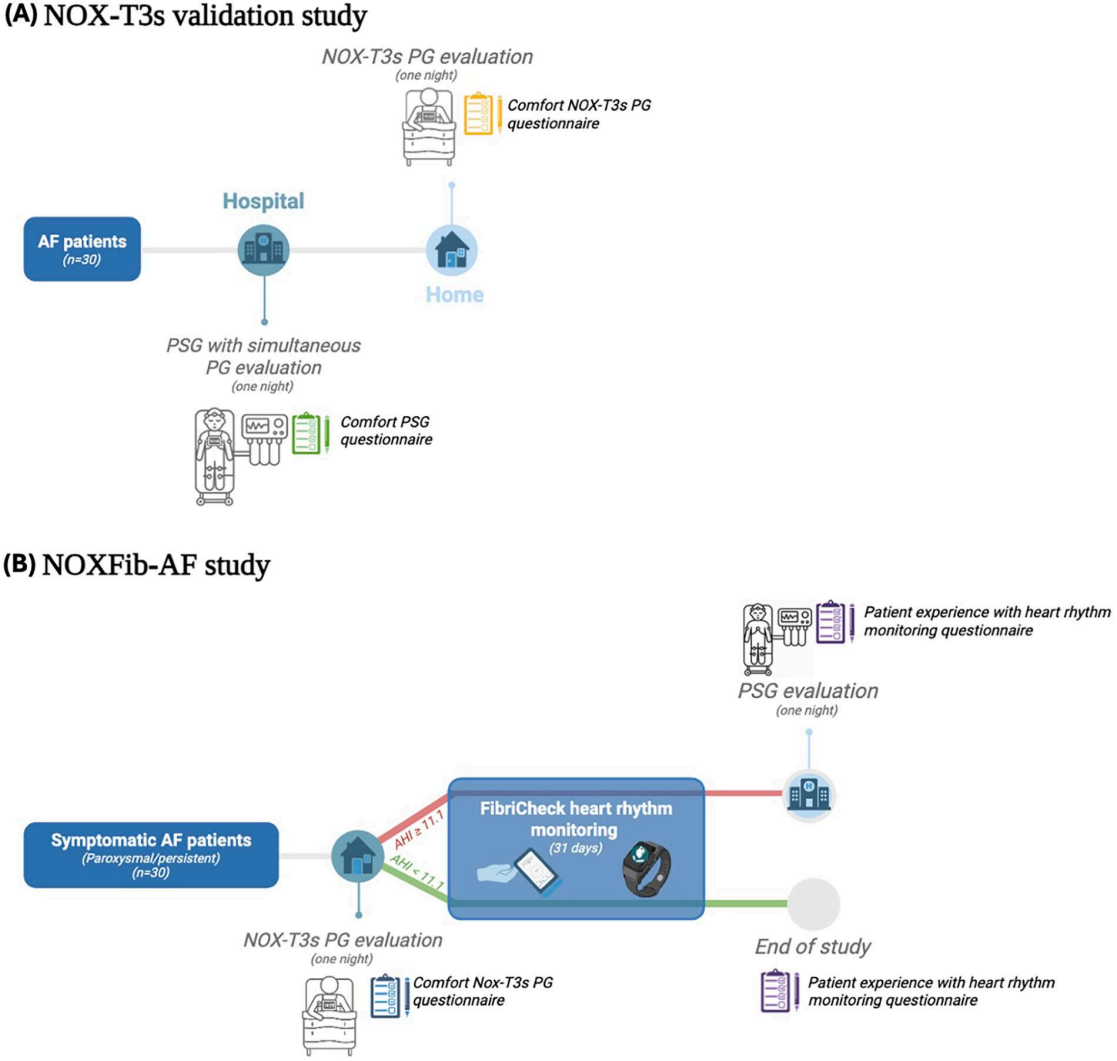
Study design NOX-T3s validation study **(A)** and NOXFib-AF pilot study **(B)** AF, atrial fibrillation; AHI, apnea-hypopnea index (events/h); OSA, obstructive sleep apnea; PG, cardiorespiratory polygraphy; PSG, polysomnography. Created with BioRender.com.

The primary outcome of this validation study was to examine the diagnostic performance and accuracy of the NOX-T3s PG device. Device specifications are described in Supplementary Table S1. User-friendliness of the NOX-T3s PG device was evaluated as a secondary outcome. Figure 1A shows the study design of this validation study, which lasted two nights. All included patients had a planned overnight PSG evaluation in the sleep clinic of UZA (Natus Schwarzer; Widdleton, USA) or Jessa (Medatec Dream; Braine-le-Château, Belgium). On the first night, participants wore the NOX-T3s PG device simultaneously with their PSG evaluation, with study staff attaching the NOX-T3s device to avoid interference with the PSG channels. The next morning, the study personnel performed a readout of the NOX-T3s and afterwards reattached the device to the patient for the next night at home. Patients only needed to connect the 3150 WristOX_2_ saturation meter (Nonin Medical; Plymouth, USA) and nasal cannula (Nox Medical, Reykjavik, Iceland) themselves before going to sleep using a detailed manual. They also logged their wake and sleep times in a diary, along with any nocturnal disturbances or activities. On both the first and second morning, participants completed a Comfort Questionnaire (CQ) to evaluate their experiences with the PSG and NOX-T3s PG evaluations, respectively (Supplementary Annex 1 and 2).

PSG recordings were manually scored by sleep clinic staff according to the American Academy of Sleep Medicine (AASM) 2012 criteria (26). The AHI was calculated as the number of apneas and hypopneas divided by total sleep time in hours. An AHI of 5 or more events per hour indicates sleep apnea, classified as follows: <5 = no sleep apnea, 5–14 = mild, 15–30 = moderate, ≥30 = severe. Sleep apnea was categorized as OSA if >50% the total events were obstructive apneas and hypopneas. For the NOX-T3s examinations, data were analyzed with the Nocturnal software using the Nox BodySleep automated analysis protocol (Nox Medical, Reykjavik, Iceland) to determine AHI values. Additionally, study personnel manually reviewed the quality of the recording (mainly focusing on nasal flow, respiratory effort, and oxygen saturation) and removed artefacts and awake periods based on the nightly activity log. If the signal quality was insufficient (i.e., <4 h of sleep, according to the AASM guidelines), the data were excluded for further analysis (26).

#### 2.2.2 NOXFib-AF study

The primary objective of NOXFib-AF was to evaluate the feasibility of implementing two ambulatory tools in patients with AF: (1) the NOX-T3s device as a screening tool for moderate-to-severe OSA, and (2) a Fitbit Versa 2 smartwatch (Google; California, USA) coupled with the FibriCheck® algorithm (Qompium NV; Hasselt, Belgium) for semi-continuous long-term heart rhythm monitoring. The secondary objective was to assess the user-friendliness of both tools. Eligible patients (distinct from those enrolled in the NOX-T3s validation study) underwent an overnight at-home OSA screening with the NOX-T3s PG device, followed by 31-day heart rhythm monitoring as outlined in Figure 1B. For the OSA screening, the study personnel attached the NOX-T3s device to the participant. All participants wore the NOX-T3s at home for one day to monitor their cardiorespiratory functions. Heart rhythm was evaluated for 24 h via a two-channel bipolar thoracic ECG. Patients only needed to connect the saturation meter and nasal cannula themselves before going to sleep, using a detailed manual. In the morning, after the NOX-T3s examination, they completed a nightly activity log and the CQ (Supplementary Annex 2). The NOX-T3s recordings were analyzed using an automated protocol and manually checked for quality, as described in the validation study above. Patients with OSA suspicion with the NOX-T3s (based on the appropriate AHI cut-off established in the NOX-T3s validation study) were advised to undergo a confirmatory in-hospital PSG examination. After OSA screening, study personnel installed the necessary applications (Fitbit® and FibriCheck®) and connected the smartwatch to the patient's smartphone for 31-day heart rhythm monitoring. The FibriCheck application automatically monitors heart rhythm using PPG. The reported sensitivity for AF detection via FibriCheck ranges from 93.4–96.3%, while the specificity for excluding AF ranges between 98.4–99.3% (17, 18). These variations depend on the applied measurement protocol, including spot-check measurements vs. (semi-) continuous smartwatch-based monitoring (17, 18). In this study, heart rhythm was measured semi-continuously via the smartwatch, with one-minute measurements performed every 9-minutes without any need for patient action. Additionally, patients were instructed to perform two spot-check heart rhythm measurements daily (morning and evening), as well as additional measurements in case of symptoms. These measurements were performed using the FibriCheck® application installed on their smartphone. The app uses the smartphone's camera and flashlight to record PPG signals from the fingertip during a one-minute measurement. The recordings from the semi-continuous monitoring and spot-check measurements are classified by the FibriCheck® algorithm as green (sinus rhythm), red (suggestive of AF), orange (mild irregularity), or blue (insufficient quality). Blue or low quality measurements are recordings with insufficient signal quality due to motion artefacts, noise, or erratic pulse waveforms, preventing reliable rhythm classification by the FibriCheck algorithm. These measurements, along with orange measurements (mild irregularity), were filtered from the dataset before analysis. Red and green recordings were used to identify AF episodes, defined as consecutive red recordings terminated by at least one green. AF burden was defined as the percentage of time spent in AF during the total monitoring period. At the end of the monitoring period, patients completed a patient experience questionnaire on heart rhythm monitoring (Supplementary Annex 3).

The feasibility of OSA screening was determined by the percentage of successful NOX-T3s recordings from initial attempts and the percentage of patients with a positive test following the advice to undergo a PSG for confirmation. In those who underwent PSG, the accuracy of the NOX-T3s device was further analyzed. For the heart rhythm monitoring, the success rate of Fitbit and FibriCheck® app installations on patients’ smartphones was assessed. Compliance was measured by the number of actual measurements taken compared to the recommended amount, while motivation was evaluated based on the percentage of days with two or more spot-checks and at least 90% of the expected semi-continuous measurements (given that there are also charging moments for the smartwatch). Additionally, the quality of the recorded heart rhythm data was examined.

### 2.3 Statistical analysis

Data from the NOX-T3s validation study were analyzed in the same way as the CarpOSAF study previously described (22). Analyses were performed using a Language and Environment for Statistical Computing [R Core Team (2023), R Foundation for Statistical Computing, Vienna, Austria] and GraphPad Prism (Boston, USA, v10.6.0). Normality was assessed with the Shapiro–Wilk test. Normally distributed data were presented as mean ± standard deviation (SD) and compared using an independent t-test or paired t-test, while non-normal data were presented as median [interquartile Range (IQR)] and compared using Mann–Whitney U tests or Wilcoxon signed-rank tests. Categorical variables were reported as percentages. Sensitivity, specificity, positive predictive value (PPV), negative predictive value (NPV) and accuracy were calculated for the cut-off value predicting the risk for clinically relevant OSA with the NOX-T3s. Agreement between NOX-T3s and PSGs (simultaneous and separate measurements) was assessed using Cohen's *κ* and Bland–Altman analysis. Pearson's r or Spearman's *ρ* were used for correlations, depending on normality. Receiver operating characteristic (ROC) curves and corresponding areas under the curve (AUC) were generated for predicting at least moderate OSA. Optimal cut-offs for the NOX-T3s were determined based on high sensitivity or specificity and Youden's J index. A *p-*value < 0.05 was considered statistically significant. As this was part of a validation and feasibility study, no specific sample size was calculated.

## 3 Results

### 3.1 NOX-T3s validation study

#### 3.1.1 Patient characteristics

A total of 30 patients, 15 of each center, with a history of AF or atrial flutter were included. The mean age was 62.5 ± 9.2 years, 86.7% were male, and the mean BMI was 30.0 ± 4.7 kg/m^2^ (Table 1). Half of the patients were diagnosed with paroxysmal AF (51.7%), and most patients were asymptomatic (mEHRA 1, 60%). Rhythm control was pursued in 13.3%, rate control 30.0%, and a combination of both in 26.7% of the patients, while 30.0% did not pursue either rate or rhythm control.

**Table 1:** Baseline characteristics of the NOX-validation study population.

Baseline characteristics	Total (*n* = 30)	Jessa Hospital (*n* = 15)	UZA (*n* = 15)	*P-*value
Age (years), mean ± SD	62.5 ± 9.2	62.2 ± 10.2	62.9 ± 8.5	*0*.*847*
Male, *n* (%)	26 (86.7)	13 (86.7)	13 (86.7)	*1*.*000*
BMI (kg/m^2^), mean ± SD	30.0 ± 4.7	29.5 ± 4.0	30.5 ± 5.4	*0*.*547*
Neck circumference (cm), mean ± SD	41.2 ± 4.0	40.9 ± 3.7	41.5 ± 4.4	*0*.*707*
Waist circumference (cm), mean ± SD	109.4 ± 15.2	109.8 ± 12.6	109.0 ± 17.9	*0*.*888*
Kind of AF, *n* (%)^a^				*0*.*434*
* De novo AF*	6 (20.7)	4 (28.6)	2 (13.3)	
* Paroxysmal AF*	15 (51.7)	8 (57.1)	7 (46.7)	
* Persistent AF*	5 (17.2)	2 (14.3)	3 (20.0)	
* Permanent AF*	2 (6.9)	0 (0)	2 (13.3)	
* Flutter*	1 (3.4)	0 (0)	1 (6.7)	
Time since AF diagnosis (years), median (IQR)^a^	5.9 (3.9–11.1)	5.2 (3.7–11.0)	6.2 (4.4–13.0)	*0*.*445*
CHA_2_DS_2_-VASc score, median (IQR)	2.0 (1.0–2.0)	2.0 (1.0–2.5)	2.0 (1.0–2.0)	*0*.*804*
HAS-BLED score, median (IQR)	1.0 (0.0–1.0)	1.0 (0.5–1.5)	1.0 (0.0–1.0)	*0*.*400*
mEHRA symptom classification, *n* (%)				*0*.*434*
* 1*	18 (60)	11 (73.3)	7 (46.7)	
* 2a*	6 (20)	2 (13.3)	4 (26.7)	
* 2b*	4 (13.3)	2 (13.3)	2 (13.3)	
* 3*	2 (6.7)	0 (0)	2 (13.3)	
* 4*	0 (0)	0 (0)	0 (0)	
Oral anticoagulation therapy, *n* (%)				*0*.*124*
* NOAC*	20 (66.7)	9 (60)	11 (86.7)	
* VKA*	2 (6.7)	0 (0)	2 (13.3)	
* None*	8 (26.7)	6 (40)	2 (13.3)	
Rate and rhythm control				
*Rhythm control, n (%)*	4 (13.3)	2 (13.3)	2 (13.3)	*1*.*000*
*Rate control, n (%)*	9 (30.0)	5 (33.3)	4 (26.7)	*0.699*
*Combination, n(%)*	8 (26.7)	3 (20.0)	5 (33.3)	0,682
*None, n(%)*	9 (30.0)	5 (33.3)	4 (26.7)	*1.000*
Underwent ECV, *n* (%)	18 (60)	8 (53.3)	10 (66.7)	*0*.*709*
Underwent PVI, *n* (%)	7 (23.3)	3 (20)	4 (26.7)	*1*.*000*
OSA severity				*0*.*608*
* No OSA*, *n* (%)	3 (10)	2 (13.3)	1 (6.7)	
* Mild OSA*, *n* (%)	9 (30)	6 (40)	3 (20)	
* Moderate OSA*, *n* (%)	11 (36.7)	4 (26.7)	7 (46.7)	
* Severe OSA*, *n* (%)	7 (23.3)	3 (20)	4 (26.7)	

AF, atrial fibrillation; BMI, body mass index; CHA2DS2-VASc: congestive heart failure, hypertension, age ≥75 years, diabetes mellitus, stroke, vascular disease, age 65–74 years, sex category; ECV, electrical cardioversion; HAS-BLED, hypertension, abnormal liver/renal function, stroke, bleeding history, labile international normalized ratio, elderly (> 65 years), Drugs/alcohol use; IQR, interquartile range; mEHRA, modified European Heart Rhythm Association; NOAC, non-vitamin K antagonist oral anticoagulant; OSA, obstructive sleep apnea; PVI, pulmonary vein isolation; SD, standard deviation; VKA, vitamin K antagonist.

a Missing data of one patient.

#### 3.1.2 Successful NOX-T3s PG registrations and comparison with the gold standard PSG

The NOX-T3s PG evaluation, performed simultaneously with the PSG in a controlled environment, was successful (i.e., ≥4 h of qualitative data), in 96.7% of participants, whereas the evaluation performed at home had a success rate of 86.7%. When comparing AHI values between the NOX-T3s and the PSG evaluation on the same night and on separate nights, no significant difference was observed [simultaneous recording *Δ*0.5 (IQR:−4.2–3.3); *p* *=* 0.942; separate-night recordings *Δ*1.5 (IQR:−3.7–7.4); *p* *=* 0.306); Table 2]. For the simultaneous in-hospital recording, there was a strong, linear, and statistically significant positive correlation (Spearman's *ρ*=0.83, *p* *<* 0.001), which remained consistent during the subsequent home recording (Spearman's *ρ*=0.68, *p* *<* 0.001) (Table 2). This suggests that the observed correlation is unlikely due to random chance. Cohen's Kappa showed a moderate diagnostic agreement across all OSA severity categories (normal, mild, moderate, and severe) of 0.60 for the in-hospital setting and a fair agreement of 0.34 for the home setting (Table 2). Bland-Altman analysis for the simultaneous recording showed a mean bias of −0.04 events/h [95% limits of agreement (LoA): −10.2 to 10.1; *p* *=* 0.963] (Supplementary Figure S1). For the home recording compared to the PSG, the NOX-T3s slightly underestimates the AHI with −2.38 events/h (95% LoA: −27.1 to 22.3; *p* *=* 0.354) (Supplementary Figure S1). Comparing the AHI values of the NOX-T3s examination in the hospital and at home, no significant difference was observed (Supplementary Table S2). Intraindividual Night-to-Night Variability (NtNV) categorical change (at AHI cut-off of 15 events/h) was seen for 8.0% of patients with the NOX-T3s (Supplementary Table S2).

**Table 2:** Comparison of apnea-hypopnea Index (AHI) between the polysomnography (PSG) and NOX-T3s polygraphy (PG).

AHI PSG	AHI PG	*Δ*	*p*-value	Spearman's *ρ*	Cohen's *κ*
Same night (*n* = 29)					
16.6 (7.8–27.4)	16.4 (11.3–27.2)	0.5 (−4.2–3.3)	0.942	0.83	0.60
Separate night (*n* = 26)					
16.4 (6.9–25.5)	14.8 (7.0–27.1)	1.5 (−3.7–7.4)	0.306	0.68	0.34

All values are presented as Median and Interquartile range (IQR). AHI, apnea-hypopnea index (events/h). The strength of the Spearman's correlation was interpreted as negligible (*ρ* = 0.00–0.10), weak (*ρ* = 0.10–0.39), moderate (0.40–0.69), strong (0,70–0,89) and very strong (0.90–1.00). The strength of the severity agreement via Cohen's κ was interpreted as no agreement (κ = 0.00), slight agreement (κ = 0.10–0.20), fair agreement (κ = 0.21–0.40), moderate agreement (κ = 0.41–0.60), substantial agreement (κ = 0.61–0.80), near perfect agreement (κ = 0.81–0.99), perfect agreement (κ = 1).

#### 3.1.3 Performance of the NOX-T3s in predicting clinically relevant OSA in patients with AF

The diagnostic performance of the NOX-T3s in detecting clinically relevant OSA (AHI≥15) during PSG and at home evaluations is summarized in Table 3. In the controlled PSG environment, the device demonstrated high accuracy, with a sensitivity of 77.8% and a specificity of 81.8% (PPV: 87.5%; NPV: 69.2%, AUC: 0.83). Performance remained comparable when used independently at the home setting, with a sensitivity of 71.4% and a specificity of 75.0% (PPV: 76.9%; NPV: 69.2%; AUC: 0.80).

**Table 3:** Diagnostic performance of the NOX-T3s PG in predicting clinically relevant (moderate-to-severe) OSA in AF patients.

*AHI* ≥ *15*	*During PSG (n = 29)*	*At home (n = 26)*
*Sensitivity (%)*	77.8	71.4
*Specificity (%)*	81.8	75.0
*PPV (%)*	87.5	76.9
*NPV (%)*	69.2	69.2
*AUC (95%CI)*	0.828 (0.679–0.978)	0.804 (0.620–0.987)
*ROC curve*	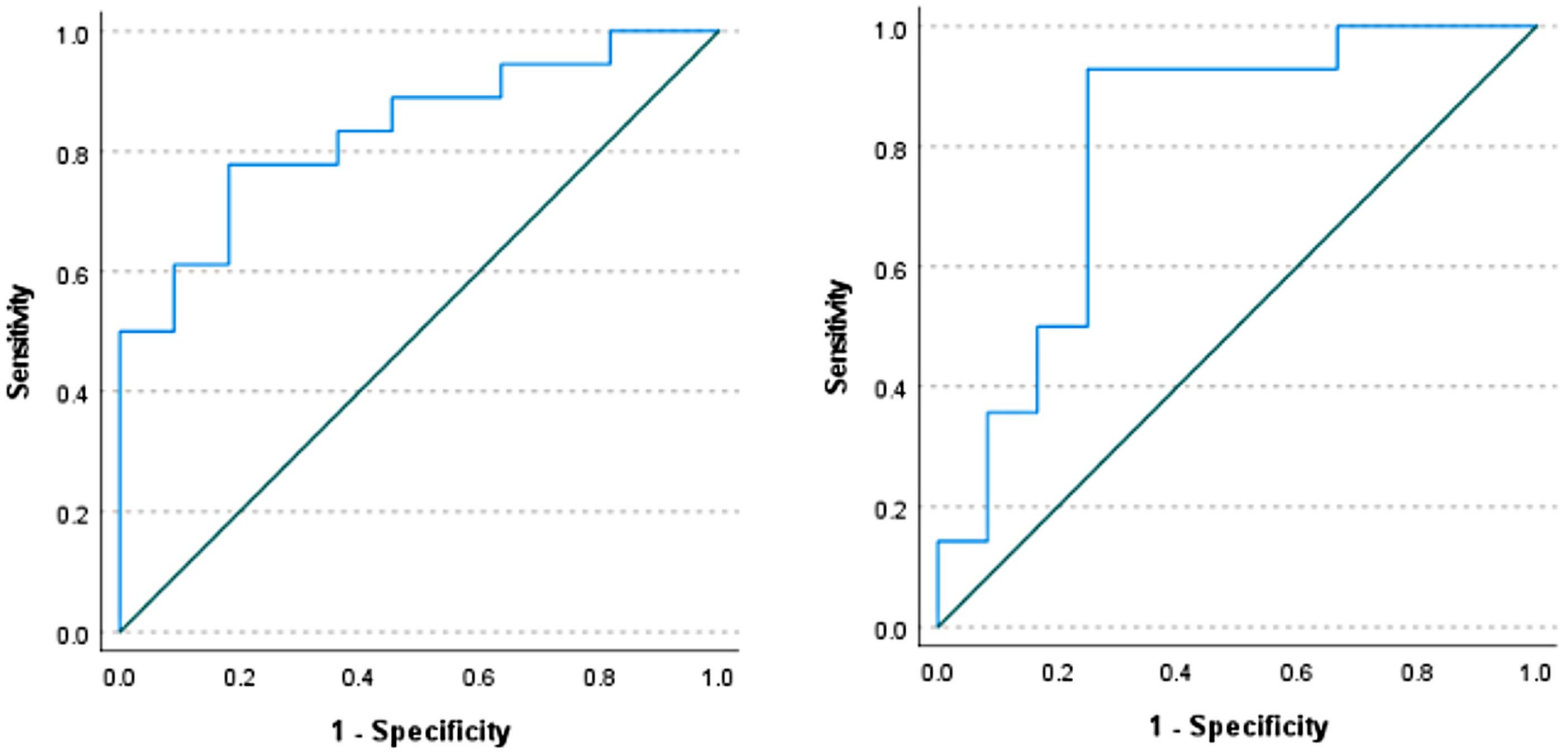	

AHI, apnea-hypopnea index (events/h); AUC, area under the curve; PPV, positive predictive value; PSG, polysomnography; NPV, negative predictive value; ROC curve, receiver operating characteristic curve; CI, confidence interval.

#### 3.1.4 Determining optimal NOX-T3s cut-off values for detecting clinically relevant OSA

The diagnostic performance parameters already demonstrated a strong discriminatory ability. However, further optimization is possible by utilizing appropriate cut-off values for the NOX-T3s based on the Youden's J index. The coordinates for the AHI cut-off values, along with their corresponding sensitivity, specificity, and Youden's J index, can be found in Supplementary Table S3. During the in-hospital examination, a cut-off AHI value of 15.35 events/h for the NOX-T3s yielded optimal specificity of 77.8%, sensitivity of 81.8%, and accuracy of 0.79. During home examinations, a cut-off AHI value of 11.1 events/h could optimize the sensitivity from 71.4% to 92.9%. Specificity remains unchanged at 75.0%, and accuracy improves to 0.85. This cut-off value was used in the NOXFib-AF study to screen for clinically relevant OSA in the home setting of the participants.

#### 3.1.5 Convenience of the NOX-T3s device

Patient satisfaction with the NOX-T3s PG device was high. The overall median comfort score was significantly higher for the NOX-T3s (9.0 IQR: 8.0–10.0) compared to the PSG (8.0 IQR: 7.0–9.0; *p* *=* 0.002). Patients reported the device was very easy to attach [median score of 10.0 (IQR: 9.0–10.0)], and experienced minimal discomfort during sleep (median score of 1.0, IQR: 0.0–2.5).

### 3.2 NOXFib-AF pilot study

#### 3.2.1 Patient characteristics

**Figure 2:**
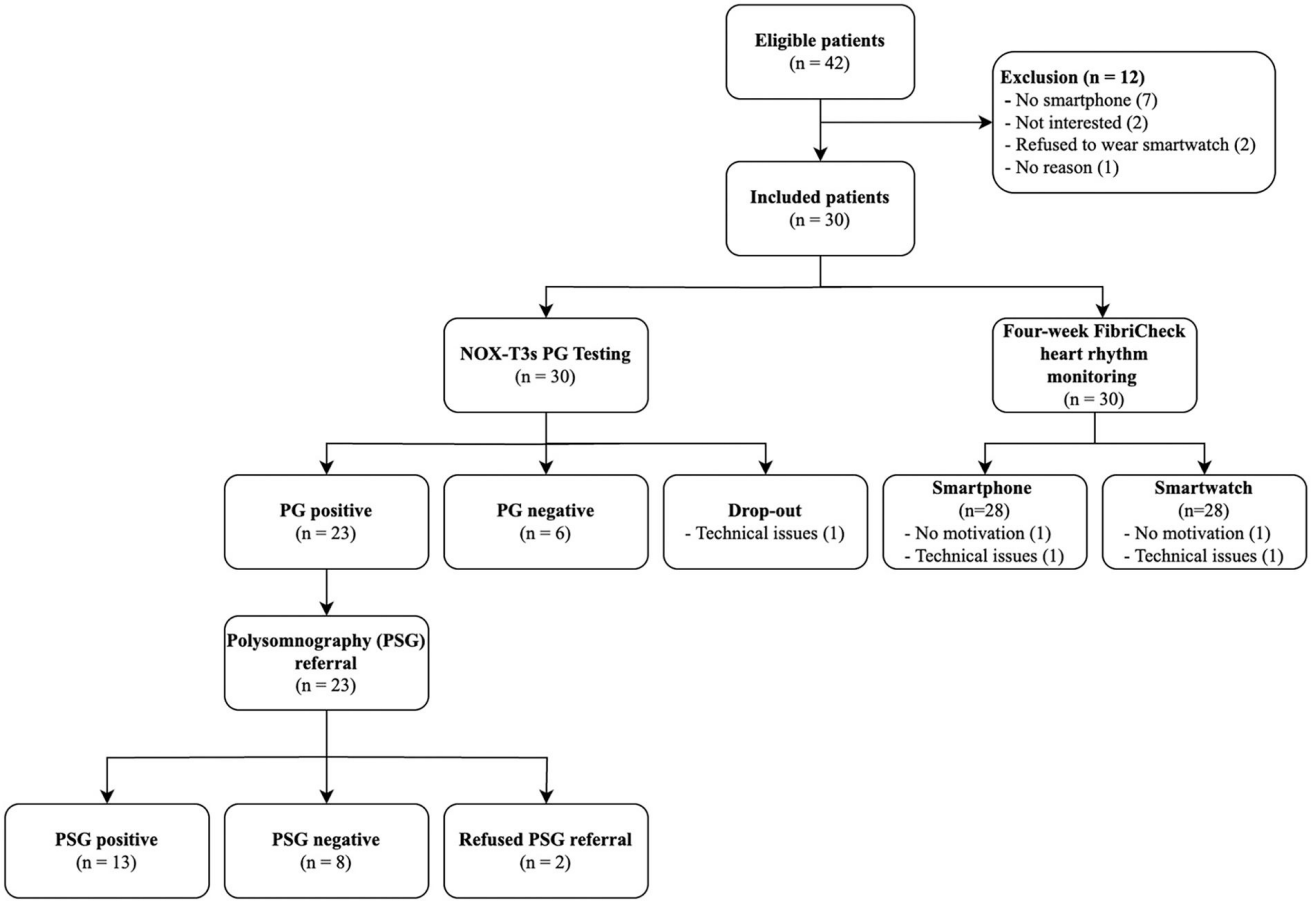
Enrolment procedure NOXFib-AF study. AF, atrial fibrillation; OSA, obstructive sleep apnea; PG, polygraphy; PSG, polysomnography.

Of the 42 patients with symptomatic AF enrolled in the study, 30 were included. Seven patients were ineligible because they did not have a smartphone, and five declined to participate (Figure 2). The included participants had a median age of 67.0 years (IQR: 60.0–72.5) and a mean BMI of 29.0 ± 5.1 kg/m^2^, with 63.3% being male (Table 4). Half of the participants experienced moderate symptoms without affecting their daily activities (mEHRA 2b; 53.3%) and 70% were diagnosed with paroxysmal AF.

**Table 4:** Baseline characteristics of the NOXFib-AF study population.

Baseline characteristics	Total (*n* = 30)	AF patients with + OSA screening (*n* = 23)	AF patients with - OSA screening (*n* = 6)	*P-*value between OSA groups
Age (years), median (IQR)	67.0 (60.0–72.5)	67.0 (60.0–74.0)	63.0 (60.0–67.5)	*0*.*224*
Male, *n* (%)	19 (63.3)	13 (56.5)	5 (83.3)	*0*.*362*
Center, *n* (%)				
* Jessa Hospital*	18 (60.0)	12 (52.2)	5 (83,3)	*0*.*354*
* UZA*	12 (40.0)	11 (47,8)	1 (16,7)	
BMI (kg/m^2^), mean ± SD	29.0 ± 5.1	29.9 ± 5.3	25.2 ± 1.8	***0***.***002***
Neck circumference (cm), median (IQR)	41.0 (38.2–42.0)	40.5 (38.5–42.0)	41.0 (38.6–41.0)	*0*.*664*
Waist circumference (cm), mean ± SD				
AF-related clinical data	106.3 ± 12.9	108.2 ± 13.6	98.9 ± 8.8	*0*.*065*
Kind of AF, *n* (%)				*1*.*000*
* Paroxysmal AF*	21 (70.0)	16 (69.6)	4 (66.7)	
* Persistent AF*	9 (30.0)	7 (30.4)	2 (33.3)	
Time since AF diagnosis (years), median (IQR)	3.4 (2.3–5.3)	3.6 (2.3–5.2)	4.3 (2.7–7.2)	*0*.*813*
CHA_2_DS_2_-VASc score, median (IQR)	2.0 (1.0–3.0)	2.0 (1.0–3.0)	1.0 (0.0–2.8)	*0*.*168*
HAS-BLED score, median (IQR)	1.0 (1.0–2.0)	1.0 (1.0–2.0)	0.8 ± 0.8	*0*.*451*
mEHRA symptom classification, *n* (%)				*0*.*211*
* 2a*	11 (36.7)	10 (43.5)	1 (16.7)	
* 2b*	16 (53.3)	11 (47.8)	4 (66.7)	
* 3*	2 (6.7)	2 (8.7)	0	
* 4*	1 (3.3)	0	1 (16.7)	
Technical aspects				
Smartphone type, *n* (%)				
* Samsung*	16 (53.3)	11 (47.8)	4 (66.7)	*1*.*000*
* iPhone*	9 (30.0)	7 (30.4)	2 (33.3)	
* Motorola*	3 (10.0)	3 (13.0)	0	
* Huawai*	1 (3.3)	1 (4.3)	0	
* OnePlus*	1 (3.3)	1 (4.3)	0	
Operating system, *n* (%)				*1*.*000*
* Android*	21 (70.0)	16 (69.6)	4 (66.7)	
* iOS*	9 (30.0)	7 (30.4)	2 (33.3)	

AF, atrial fibrillation; BMI, body mass index; OSA, obstructive sleep apnea; CHA_2_DS_2_-VASc, congestive heart failure, hypertension, age ≥ 75 years, diabetes mellitus, stroke, vascular disease, age 65–74 years, sex category; HAS-BLED, hypertension, abnormal liver/renal function, stroke, bleeding history, labile international normalized ratio, elderly (> 65 years), Drugs/alcohol use; mEHRA, modified European Hearth Rhythm Association; IQR, interquartile range; SD, standard deviation. Bold indicates significant *p*-values <0.05.

#### 3.2.2 Feasibility of ambulatory at home OSA screening with NOX-T3s in routine clinical practice

**Figure 3:**
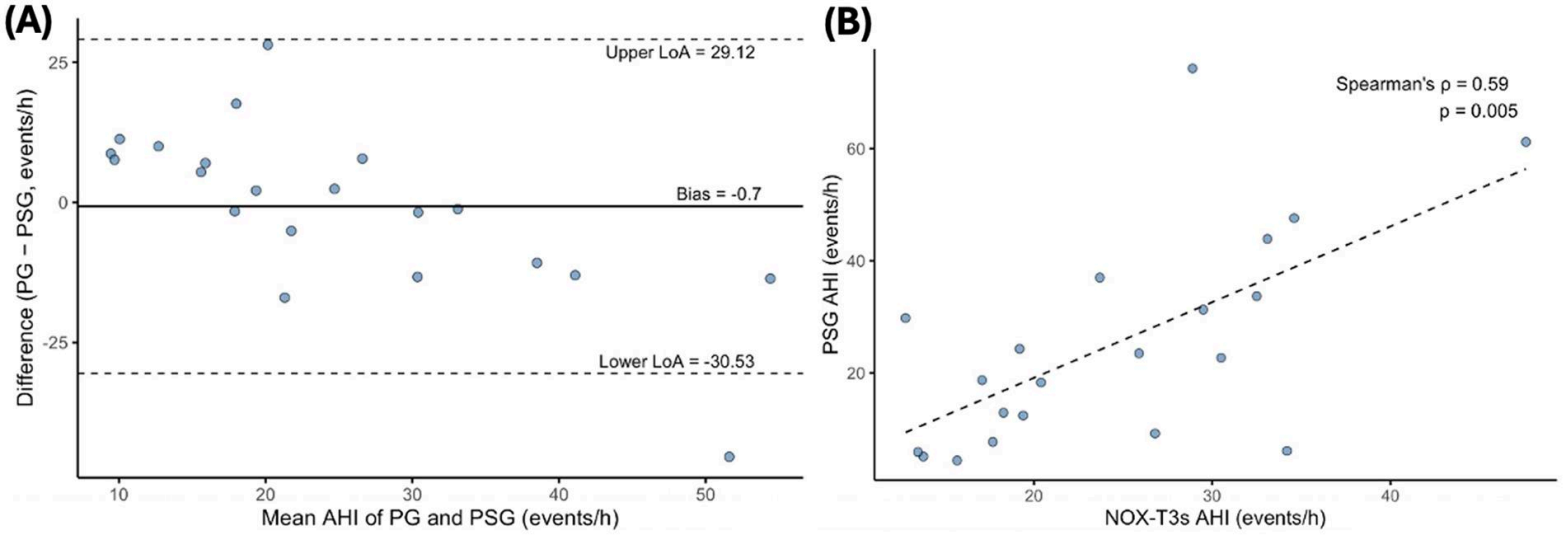
Agreement between the AHI derived from the PG and PSG evaluation illustrated by a bland–altman plot (*n* = 21) **(A)** and scatterplot of the AHI values obtained from the simultaneous PG and PSG evaluation (*n* = 21) **(B)** during the NOXFib-AF study. The strength of the Spearman's correlation was interpreted as negligible (*ρ* = 0.00-0.10), weak (*ρ* = 0.10-0.39), moderate (*ρ* = 0.40-0.69), strong (*ρ* = 0,70-0,89) and very strong (*ρ* = 0.90-1.00). Each dot represents one participant. AHI, apnea-hypopnea index (events/h); PG, cardiorespiratory polygraphy; PSG, polysomnography; LoA, limits of agreement.

Of the 30 NOX-T3s PG examinations, 26 (86.7%) were successful on the first attempt, with a median signal quality of 98.5% (IQR: 95.5–100). Four participants had an unsuccessful first examination. Of the unsuccessful NOX-T3s PG recordings, one was prematurely terminated (no overnight recording), one showed a poor blood oxygen saturation signal, one a poor nasal flow signal, and one showed poor signal quality for both signals. Three of these agreed to take a new test, which was successful, resulting in an overall success rate of 97.1% of all performed NOX-T3s PG evaluations (*n* = 34). Clinically relevant OSA, as determined by an AHI≥11.1 based on the NOX-T3s validation study, was detected in 79.3% of patients. The median sleep efficiency, calculated as the total sleep time divided by total time in bed, was 88.4% (IQR: 81.3–90.7). Participants who screened positive for OSA had a significantly higher BMI than those who screened negative (29.9 ± 5.3 kg/m^2^ vs. 25.2 ± 1.8 kg/m^2^, *p* *=* 0.002; Table 4)**.** Of the 23 positive screenings, 21 (91.3%) followed the advice to undergo a PSG examination; one was not open to CPAP treatment if the PSG would be positive, and the other refused the PSG examination. PSG was performed 104 ± 45 days after the screening test. The NOX-T3s had a positive predictive value of 61.9%, as 13 of the 21 patients were subsequently diagnosed with moderate-to-severe OSA via PSG in the sleep clinic. One patient (4.7%) had no OSA (AHI < 5), seven patients (33.3%) were classified as mild OSA (AHI: 5–14), six (28.6%) as moderate OSA (AHI: 15–30) and seven (33.3%) as severe OSA (AHI≥30). Bland-Altman analysis showed a slight, not statistically significant, underestimation of the AHI by the NOX-T3s (−0.7 events/h, *p* *=* 0.834), with LoA ranging from −30.5 to 29.1 events/h compared to PSG (Figure 3). A moderate positive correlation was found between the AHI values obtained from NOX-T3s and PSG (*ρ*=0.59; *p* = 0.005) (Figure 3).

#### 3.2.3 Feasibility of the implementation of semi-continuous smartwatch-based heart rhythm monitoring in routine clinical practice

**Figure 4:**
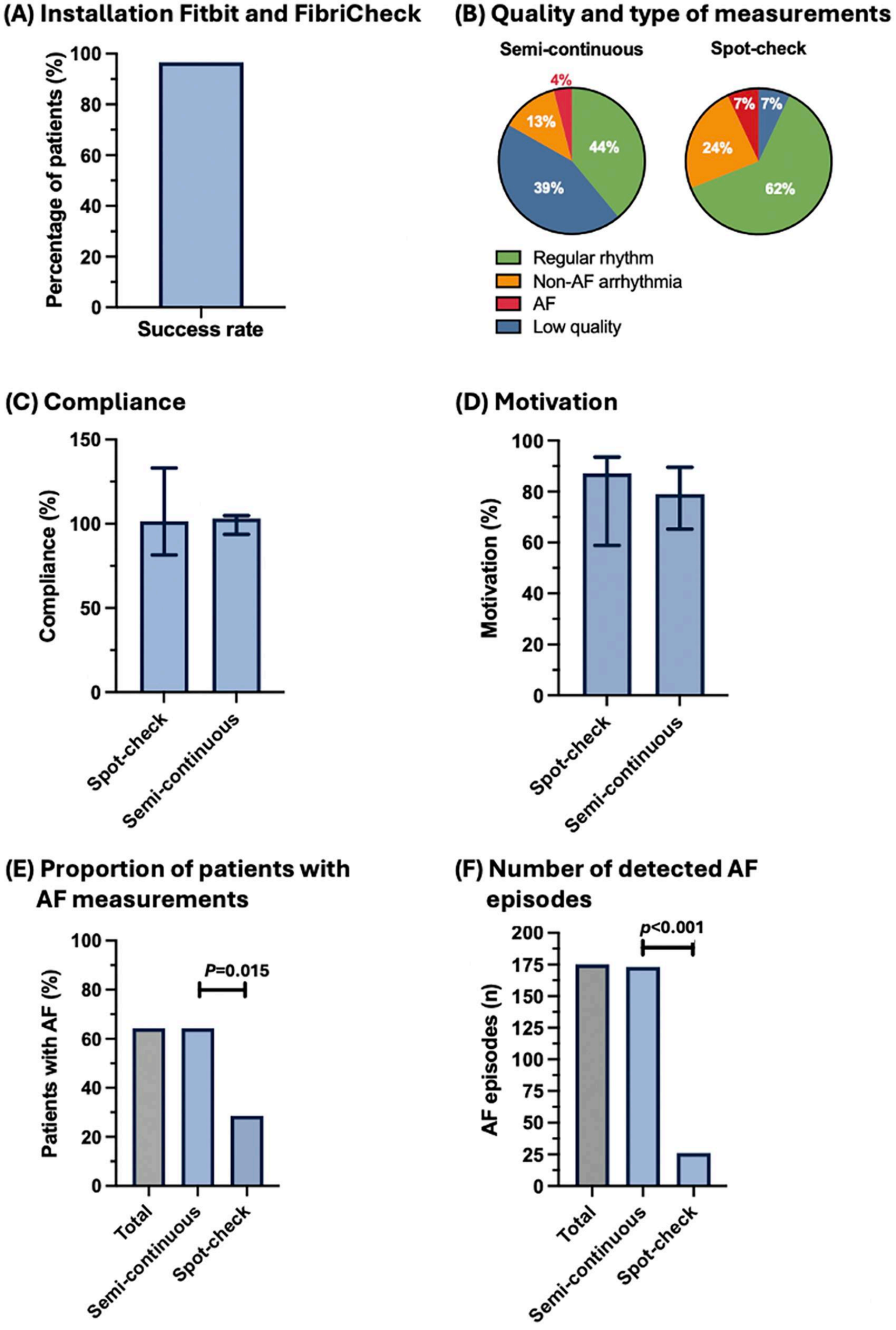
Feasibility of implementing semi-continuous and spot-check heart rhythm monitoring. **(A)** Success rate of Fitbit and FibriCheck application installation; **(B)** Quality and type of semi-continuous and spot-check measurements; **(C)** Compliance with each monitoring method; **(D)** Motivation for each monitoring method; **(E)** Proportion of patients in whom AF was detected in total dataset (semi-continuous and spot-check combined), by semi-continuous monitoring, and by spot-check monitoring; and **(F)** number of AF episodes detected in total dataset (semi-continuous and spot-check combined), by semi-continuous monitoring, and by spot-check monitoring. Data are presented as median (IQR) due to the non-normal distribution of the data. AF, atrial fibrillation; IQR, interquartile range. A p-value < 0.05 was considered statistically significant.

The Fitbit device, coupled with the FibriCheck application, was successfully installed in 96.6% of the participants (Figure 4A). The median monitoring duration was 31 days (IQR:30–31). Of the semi-continuous smartwatch data, 61% ± 11.5 were of high quality and therefore clinically useful, with a median of 70 (IQR: 65.5–84.5) high-quality measurements per day and a mean of 2,126 ± 592 high-quality semi-continuous measurements over 31 days (Figure 4B). This included 44.0% of measurements indicating regular heart rhythm, 12.7% indicating non-AF arrhythmias, and 3.9% indicating AF (Figure 4B). For the spot-check protocol, a median of 63 (IQR: 51–81) measurements were performed per patient over 31 days, with only a small percentage of the measurements (3.5% (IQR: 1.6–8.0)) of low quality (Figure 4B). High-quality measurements included 62.0% of measurements indicating regular heart rhythm, 23.6% indicating non-AF arrhythmias, and 7.3% indicating AF (Figure 4B). Compliance, defined as the number of measurements performed divided by the recommended measurements (i.e., 62 for the spot-check and 90% of the expected 4,960 semi-continuous measurements, accounting for necessary smartwatch charging periods), was 101.6% (IQR:81.5–133.1) for the spot-check and 103.1% (IQR: 93.7–104.9) for the semi-continuous smartwatch monitoring protocol (Figure 4C). Values exceeding 100% indicate that some participants performed more spot-check measurements than the recommended minimum of two per day, and that smartwatch charging time was limited (i.e., <10% of total monitoring time), with participants typically resuming monitoring immediately after recharging the device. Motivation, calculated as the proportion of days with ≥2 daily spot-checks and ≥90% of the expected daily 160 semi-continuous measurements, was 87.1% (IQR: 58.9–93.6%) for the spot-check and 79.0% (IQR: 65.3–89.5%) for the semi-continuous method (Figure 4D). When considering all data (both semi-continuous and spot-check), AF was detected in 18 patients (64.3%), with a median AF burden, defined as the percentage of time spent in AF over the total monitoring duration, of 0.8% (IQR: 0.03–2.10) (Figure 4E). A total of 175 AF episodes were detected (Figure 4F). When spot-check and semi-continuous smartwatch measurements were analyzed separately, AF was detected in 64.3% of patients using the semi-continuous method, compared to 28.6% with spot-checks (*p* *=* 0.015) (Figure 4F). The semi-continuous method identified 6.7 times more AF episodes than the spot-check method (173 vs. 26; *p* *<* 0.001) (Figure 4G).

#### 3.2.4 Patient satisfaction with OSA screening and heart rhythm monitoring

**Figure 5:**
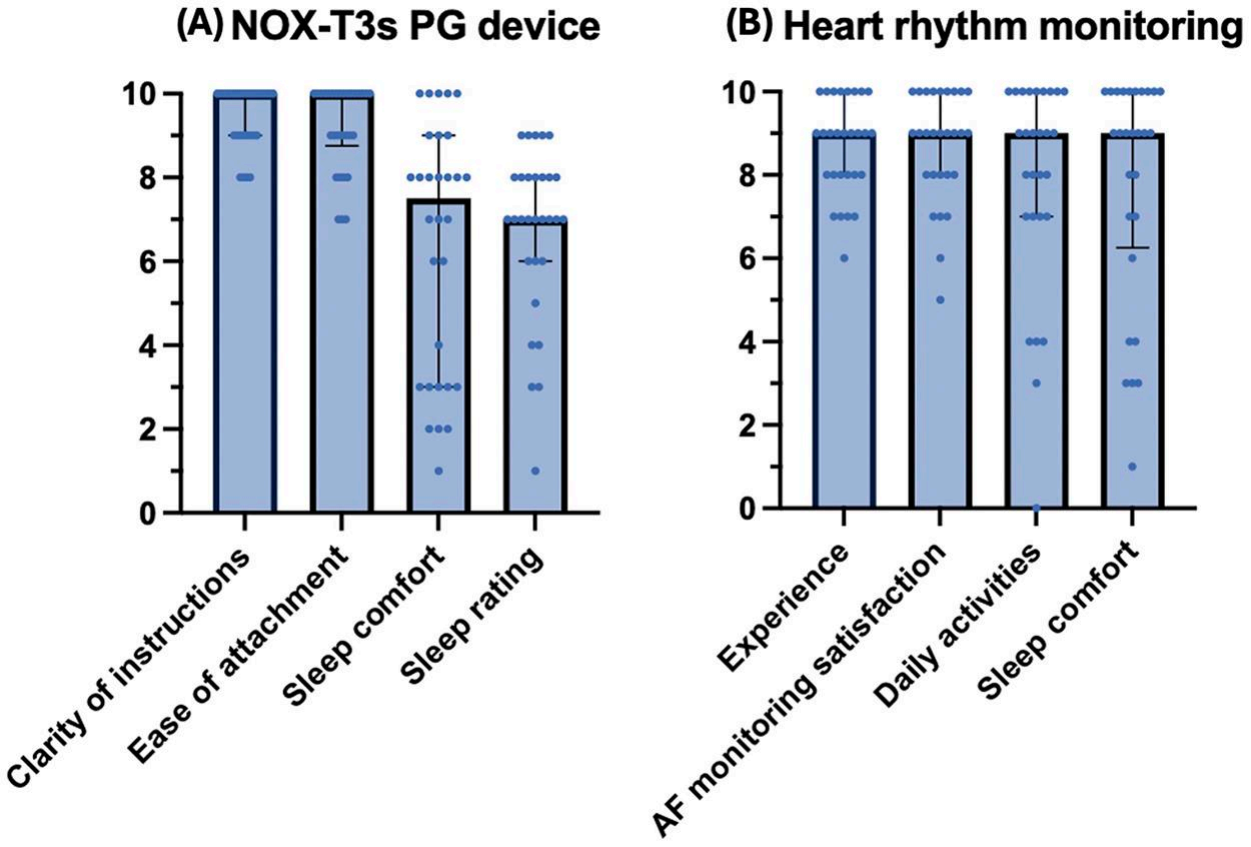
User-friendliness of **(A)** the NOX-T3s polygraphy device for OSA testing (*n* = 28) and **(B)** semi-continuous FibriCheck monitoring on fitbit versa 2 (*n* = 28) in AF patients of the NOXFib-AF pilot study. The scores are presented as patients’ median (and IQR) due to the non-normal data distribution. The dots represent the individual scores. AF, atrial fibrillation; IQR, interquartile range; OSA, obstructive sleep apnea.

Patient satisfaction was high for the ambulatory NOX-T3s PG screening [median score: 32 out of 40 (IQR: 29–35)] and semi-continuous heart rhythm monitoring [median score: 34 out of 40 (IQR: 29–37)]. The NOX-T3s was found to be relatively comfortable to sleep with (median score: 7.5, IQR: 3–9), and patients scored moderately positive on their overall night rest (median score: 7, IQR: 6–8) (Figure 5A). They also had a good experience with Fitbit-based FibriCheck monitoring (median score: 9, IQR: 8–10) and were satisfied with the follow-up of their AF (median score: 9, IQR: 8–10). Importantly, patients did not experience any hindrance to their daily activities (median score: 9, IQR: 7–10), nor did the smartwatch hinder them at night (median score: 9, IQR: 7–10) (Figure 5B). Six patients (21%) reported irritation from the Fitbit bracelet. Ten patients (35%) indicated that they would see themselves wearing the smartwatch for an indefinite period.

## 4 Discussion

These studies provided key insights. First, we validated the diagnostic performance of the NOX-T3s PG device with an automated AHI algorithm to detect clinically relevant OSA in patients with AF. Second, we demonstrated that a structured ambulatory OSA screening pathway, using the NOX-T3s with an appropriate AHI cut-off value for clinically relevant OSA, combined with smartwatch-based semi-continuous heart rhythm monitoring, is feasible, well accepted by patients, and implementable in routine clinical practice.

### 4.1 Validation and clinical implementation of the NOX-T3s for OSA screening in AF

Although PG is currently discouraged as a standalone diagnostic tool for OSA in patients with cardiorespiratory disorders, evidence in this specific context remains limited. In line with previous work by Aurora et al. in hospitalized heart failure patients and Delesie et al. in AF patients, our findings demonstrate that PG can provide reliable screening results in the AF population (22, 27). As shown in the NOX-T3s validation study, the NOX-T3s PG demonstrated good diagnostic performance for detecting moderate-to-severe OSA in patients with AF compared to the gold-standard PSG: 1) AHI values derived from simultaneous and separate-night NOX-T3s recordings did not differ significantly from those obtained from PSG measurements; 2) diagnostic accuracy was high with AUC exceeding 0.80 3) Blant-Altman analysis in both studies showed an acceptable agreement between the NOX-T3s device and PSG. During simultaneous recordings, there was a minimal bias of −0.04 events/h, whereas during home NOX-T3s recordings a slightly larger underestimation of −2.38 events/h was observed. This likely reflects differences in recording environment and the known NtNV, which resulted in a categorical change in 8.3% of patients. In addition, PG devices often underestimate the AHI because they may dilute events over a longer recording time when the actual sleep time is not fully known and they may miss events that require electroencephalogram-based detection of arousal-related hypopneas according to the AASM guidelines (26). Comparable bias and LoA have previously been reported in a study validating the NOX-T3s during both in-laboratory PSG and at home recordings in patients with cardiorespiratory comorbidities, despite expert technicians manual scoring whereas the present study relied on automated scoring together with a quick quality check (24). Importantly, prior research has demonstrated very strong agreement in AHI between automated algorithms, including the Nocturnal automatic scoring algorithms, and experienced international technologists, supporting the validity of automated scoring approaches (23). While current guidelines still recommend manual scoring, automatic scoring, which can generate a report in only 15 min without requiring expert technician input, can substantially improve testing efficiency and facilitate timely referral for confirmatory PSG and subsequent treatment when OSA is detected by PG (28). This is particularly relevant in a cardiology setting, where PG is intended to be implemented as a screening tool and can be applied by non-sleep-specialized technicians and health care providers following a brief training to attach, program and read out the device, without the need for manual scoring. This can substantially reduce workload in the sleep clinic and facilitates large scale OSA screening within the AF population.

The high diagnostic accuracy found in the NOX-T3s validation study (AUC of 0.83 for simultaneous and 0.80 for home-based recordings) is consistent with our previous validation study in patients with AF, in which three PG devices (ApneaLink Air, SOMNOtouch RESP, and SpiderSAS) showed good performance in detecting OSA (*n* = 130) (22). Importantly, optimization of the AHI cut-off using Youden's J index further improved diagnostic performance. During home-based recording, which is an essential aspect for the implementation of the NOX-T3s as an ambulatory screening strategy, the optimized AHI cut-off of 11.1 events/h improved accuracy from 0.80 to 0.85 and sensitivity from 71.4% to 92.9% without compromising specificity (75%), outperforming those reported by Delesie et al. (22). This threshold reduces the risk of missing patients with clinically relevant OSA without substantially increasing unnecessary referrals for confirmatory PSG referrals. In addition to its diagnostic performance, the success rate for the NOX-T3s in controlled hospital environment was 96.7%. While in home environment in the NOX-T3s validation study and NOXFib-AF study it was identical at 86.7%. This is higher than those observed in the previous validation study (ApneaLink Air 72%, SpiderSAS 73%, SOMNOtouch RESP 79%) and comparable with existing literature (24, 29). Although the devices were applied identically in both hospital and home settings, the lower success rate in the home environment may be explained by differences in sleep conditions and patient behavior. In the sleep lab, concurrent PSG monitoring with multiple sensors may restrict movement and enhance signal stability. At home, more natural and deeper sleep may involve increased movement and risk of sensor displacement (e.g., nasal cannula or pulse oximeter) and signal loss (22).

In our second NOXFib-AF study, we applied the NOX-T3s as an initial home-based screening tool within a structured screening pathway to evaluate whether it can effectively and efficiently identify patients who are most likely to benefit from confirmatory PSG in routine clinical practice. Symptomatic patients with paroxysmal or persistent AF who owned a compatible smartphone and were willing to participate in a digital health monitoring program were selected, as they are most likely to benefit from rhythm control strategies according to the current ESC guidelines (15). Patients with permanent AF were excluded because rhythm control is no longer pursued in this population, which limits the potential clinical impact of OSA treatment on AF-related outcomes (15). Patient participation was high, with only a small proportion declining screening (11.9%). Furthermore, only a minority (16.7%) of the contacted patients were ineligible because they did not own a smartphone. Importantly, most patients with a positive NOX-T3s PG result adhered to the recommendation for confirmatory PSG (91.3%), and OSA was confirmed in 61.9% of cases. The overall prevalence of OSA in our study was 43.3% based on the NOX-T3s and PSG results. This is comparable to the existing literature, which reports a prevalence of moderate-to-severe OSA of 42.1–56.1% in patients with AF (3, 4, 30, 31). The agreement between the NOX-T3s PG device and PSG was moderate and slightly lower compared to the validation study. The Bland–Altman analysis demonstrated a minimal mean bias of −0.7 events/h, indicating a slight underestimation of event frequency compared with the reference method. This degree of underestimation is unlikely to meaningfully affect clinical categorization in most patients, particularly when diagnostic decisions are based on clinically relevant severity thresholds rather than small numerical differences. The lower observed agreement between NOX-T3s and PSG in the NOXFib-AF study compared to the validation study is likely partly explained by NtNV, as PSG was performed months after the NOX-T3s examination rather than on a consecutive night. In addition to temporal variability, NtNV and OSA severity may also reflect differences in sleep environment and sleep behaviour, as some patients may sleep better at home than in the sleep clinic, and differences in body position between recordings may have influenced the results. Furthermore, OSA severity can fluctuate over time due to changes in weight, medication use, cardiovascular status, lifestyle factors or disease progression (32). However, this time interval between initial NOX-T3s screening and PSG reflects the real-world waiting times currently encountered in a Belgian setting at the sleep clinics of both Jessa and UZA hospital, providing valuable insights into the practical feasibility of implementing such a screening pathway in routine clinical care. Furthermore, the sample size was small, as only patients with a positive test (*n* = 21) were referred for PSG evaluation. Of these patients, only one (5%) did not meet PSG criteria for OSA, while 7 patients (33%) were diagnosed with mild OSA, several of whom were close to the diagnostic cut-off. Finally, the NOX-T3s cut-off used in our study was determined based on a single-night PG evaluation, whereas repeated evaluations over several nights (two to three nights) could further optimize diagnostic performance (33–35). This aligns with a broader shift toward longitudinal, patient-centered monitoring using accessible and validated technologies, which may better capture variability in sleep-disordered breathing compared to single-night, hospital-based assessments. Furthermore, the NOX-T3s achieved high patient comfort scores [NOX-T3s validation study: 9.0 on 10.0 (IQR: 8.0–10.0) and NOXFib-AF study: 32 out of 40 (IQR: 29–35)] compared to the other PG devices (ApneaLink Air 8.25 ± 1.42, SpiderSAS 7.97 ± 1.39, SOMNOtouch RESP 6.63 ± 1.90). However, in the study by Delesie et al., patients were required to use three different PG devices on three consecutive days, which may have influenced the success rate and patient-reported comfort scores (22).

### 4.2 Feasibility of implementing semi-continuous heart rhythm monitoring

The NOXFib-AF pilot study evaluated the feasibility of longitudinal assessment of AF recurrence and burden using semi-continuous heart rhythm monitoring with additional daily spot-check measurements. This innovative approach is essential for evaluating the potential impact of OSA diagnosis and treatment on AF outcomes, such as AF burden and recurrence, in future larger studies. Semi-continuous heart rhythm monitoring via a smartwatch, combined with the validated, CE-certified FibriCheck algorithm, is an innovative approach to address the limitations of current non-invasive rhythm monitoring modalities: 1) it enables long-term (several months to years) monitoring with high sampling frequency beyond the duration of standard Holter recordings, 2) measurements are automatically acquired at predefined intervals and do not rely on patient initiation, as is the case for handheld devices and smartphone-based applications, 3) it provides standardized, protocol-driven monitoring intervals throughout the entire monitoring period, in contrast to wearable devices that rely on intermittent PPG recordings during rest periods and typically increase sampling frequency only after suspected AF detection. In this study, smartwatch installation was successful in the majority of patients (96,6%), including the elderly, and was associated with high levels of patient compliance [103.1% (IQR: 93.7–104.9)], motivation [79.0% (IQR: 65.3–89.5%)], and comfort [median score: 34 out of 40 (IQR: 29–37)]. Notably, both overall compliance and adherence were high in our study. However, this is likely due to the relatively short 31-day monitoring period. Prior work using the same monitoring approach has demonstrated a significant decline in the number of semi-continuous measurements after approximately 2 months, suggesting that longer monitoring periods may be associated with reduced adherence over time (36). Although a proportion of the recordings were classified as low quality (39%), this was expected, given the presence of motion artefacts and the patient's unawareness of the measurements being taken by the smartwatch. This proportion of low-quality recordings is comparable to that reported in a previous study using the same monitoring strategy, which found that 41% of measurements were of low-quality (36). When comparing the twice-daily smartphone-based spot-check measurements with the semi-continuous smartwatch monitoring, the latter detected substantially 6.7 times more AF episodes and identified a higher proportion of patients with AF (64.3% vs. 28.6%) during the one-month monitoring period, as expected given its higher sampling frequency and more continuous data capture. However, this finding contradicts the results of Wouters et al., who reported that more measurements suspicious for AF were detected in the smartphone group (twice-daily spot-checks and in case of symptoms) compared to the smartwatch group (one-minute measurements every 3 min) within a 6-month monitoring period, despite using the same FibriCheck-based method (36). This may be attributed to differences in patient populations, as the patients in their study were those with cryptogenic stroke or transient ischemic attack patients who did not have a prior AF diagnosis (36). Despite including symptomatic patients, a substantial number of asymptomatic AF episodes were captured by semi-continuous monitoring in our cohort. These findings underscore the feasibility and added value of semi-continuous smartwatch-based rhythm monitoring for longitudinal assessment of AF burden and recurrence in clinical research settings. It is important to note that semi-continuous rhythm monitoring should not replace conventional ECG diagnoses but should be viewed as a complementary tool that facilitates extended rhythm surveillance over time.

### 4.3 Structured interdisciplinary care pathway for OSA detection in AF patients

The findings of this study, including key implementation aspects such as feasibility, data quality, data analysis and translation, patient adherence, acceptability, and patients’ willingness to participate in such a digital health care program, were instrumental in the design of the ongoing prospective pre-post implementation STAROSA study (NCT06263608). In this study, we aim to evaluate the effect of systematic OSA screening and treatment pathway on AF burden, defined as the proportion of patients with AF, AF recurrence rate, and time spent in AF over the monitoring period. Home-based screening will be performed using the NOX-T3s device in paroxysmal and persistent AF patients, applying the optimal cut-off value of 11.1 (determined in the NOX-T3s validation study) to identify patients with clinically relevant OSA. Patients with a positive screening result will undergo confirmatory in-hospital PSG. The waiting period for PSG will serve as a baseline rhythm-monitoring phase of three months, during which AF burden will be assessed using the FibriCheck application for twice-a-day spot-check recordings, combined with semi-continuous monitoring via smartwatch-based 9-minute interval measurements. Following PSG-confirmed OSA diagnosis, patients will initiate CPAP therapy, after which a second three-month monitoring phase will be conducted to evaluate changes in AF burden after treatment initiation.

If the STAROSA study demonstrates favorable outcomes, this approach may support the development of a structured interdisciplinary care pathway for OSA detection in AF patients, characterized by close collaboration between patients, the AF clinic, and the sleep clinic. In this model, screening would be initiated in the AF clinic and managed by specialized AF nurses in collaboration with cardiologists. Nurses would provide patients with one or more validated digital monitoring tools (e.g., NOX-T3s and smartwatch with FibriCheck), depending on clinical indication. Patients would use these devices at home and return them to the clinic for analysis by trained AF nurses or technologists. Patients with evidence suggestive of clinically relevant OSA would be referred to the sleep clinic for confirmatory PSG, which is required in Belgium for CPAP reimbursement. In patients requiring CPAP therapy, smartwatch-based monitoring would be continued to assess the impact of treatment on AF burden and sleep-related outcomes. Follow-up would be performed by a multidisciplinary team involving both AF and sleep clinics, enabling coordinated patient-centered care, including education, motivational interviewing, and support for self-management strategies such as CPAP adherence and weight management. This collaborative pathway allows bidirectional communication between clinics, facilitating timely management of both AF and OSA-related issues. Ultimately, this may reduce existing barriers to systematic OSA screening in AF populations. However, this proposed care pathway requires formal evaluation before implementation in routine clinical practice.

### 4.4 Strengths, limitations and future prospects

The strength of both studies lies in its evaluation of the diagnostic accuracy and real-world feasibility of PG for detecting moderate-to-severe OSA, and evaluating unobtrusively AF recurrence and burden in patients with AF. By integrating a validation and feasibility study across two centers, our design reflects routine clinical practice, including inter-scorer variability in PSG interpretation. Additionally, patient-reported questionnaires were used to evaluate user-friendliness. Importantly, patients had the option to consult a sleep clinic following a positive screening result, and most of them did, highlighting the acceptability and clinical relevance of this approach. Several limitations should be acknowledged. Participants in the first study were recruited from patients with AF already referred for in-hospital PSG, representing a population with a likely high pretest probability of OSA. Additionally, the measurement order was not randomized, as all patients underwent simultaneous PSG and NOX-T3s examination, followed by a consecutive night of NOX-T3s evaluation. This may have introduced a habituation effect after the first night of testing, as patients are known to sleep worse during initial recordings in a sleep laboratory and may influenced PG comfort scores. Furthermore, the relatively small sample sizes in both studies limit the generalizability of the findings. The optimal NOX-T3s AHI cut-off value of 11.1 identified for home-based OSA screening was derived from a relatively small cohort and should ideally be confirmed in a larger, independent external population before it can be broadly implemented in clinical practice. In addition, the NOXFib-AF inclusion criteria to own a smartphone and willingness to participate in a digital monitoring program may have introduced selection bias, limiting generalizability to less digitally experienced or engaged patients. Moreover, the impact of OSA diagnosis on subsequent patient management and AF-related outcomes was not assessed within the present study. As such, we were unable to evaluate downstream clinical endpoints, including CPAP compliance, changes in AF management, or the effect of OSA treatment on AF burden and symptoms. This represents an important limitation, as the ultimate value of opportunistic screening depends on its ability to translate into effective therapeutic interventions and improved patient outcomes. This will occur in the ongoing STAROSA study (NCT06263608), a prospective pre-post implementation study designed to evaluate the impact of a structured testing and treatment program for OSA using the NOX-T3s device and a Fitbit smartwatch with the FibriCheck algorithm on the AF (symptom) burden in an AF population.

## 5 Conclusions

Home-based NOX-T3s polygraphy using an automated AHI algorithm and a population-specific AHI cut-off value provides a reliable, feasible, and patient accepted approach as an initial screening step in an OSA care pathway of AF patients at the cardiology department. This strategy enables timely identification of patients for a confirmatory PSG and efficient use of PSG resources, thereby facilitating integration of OSA screening into routine AF care pathways. Importantly, this approach complements rather than replaces in-hospital PSG as diagnostic gold standard. In addition, smartwatch-based semi-continuous rhythm monitoring proved highly feasible, with high data yield, patient adherence, and comfort, supporting its use for longitudinal assessment of AF burden and recurrence. Together, these findings support a patient-centered screening and monitoring strategy that may enable future studies to evaluate the impact of OSA diagnosis and treatment on clinical outcomes in AF.

## Data Availability

The raw data supporting the conclusions of this article will be made available by the authors, without undue reservation.
