# Supplementary material for: Validation and implementation of ambulatory obstructive sleep apnea polygraphy screening combined with wearable semi-continuous heart rhythm monitoring in patients with atrial fibrillation: a validation and a pilot study
Source: Front Cardiovasc Med. 2026 Jun 26;13:1873057. doi: 10.3389/fcvm.2026.1873057 (PMC13349795; doi:10.3389/fcvm.2026.1873057)
Supplement: Supplementary file 1 [file Datasheet1.pdf]

Supplementary Material

Validation and implementation of ambulatory obstructive sleep apnea polygraphy screening combined with wearable semi-continuous heart rhythm monitoring in patients with atrial fibrillation: a validation and a pilot study

Anouk Delaet[1,2,4], Rana Önder[1], Paulien Vermunicht[3,4],Lieselotte Knaepen[1,2], Michiel Delesie[4,7], Johan Verbraecken[5], Karolien Weytjens[6], Paul Dendale[1,2], Johan Vijgen[1,2], Hein Heidbuchel[1,3,4], Lien Desteghe[1,2,3,4,8]

[1]Faculty of Medicine and Life Sciences, Hasselt University, Hasselt, Belgium, [2]Department of Cardiology, Jessa Hospital, Hasselt, Belgium [3]Research Group Cardiovascular Diseases, University of Antwerp, Antwerp, Belgium, [4]Department of Cardiology, Antwerp University Hospital, Antwerp, Belgium, [5]Multidisciplinary Sleep Disorders Center, Antwerp University Hospital and University of Antwerp, Edegem, Belgium, [6]Sleep Center Hasselt, Jessa Hospital, Hasselt, Belgium, [7]AZ Sint-Lucas Ghent, Ghent, Belgium, [8]Center for Research and Innovation in Care (CRIC), Department of Nursing and Midwifery Sciences, University of Antwerp, Antwerp, Belgium

1 Supplementary Tables

Supplementary Table S1: Specifications of the NOX-T3s

Measurements	NOX-T3s (Nox Medical)
	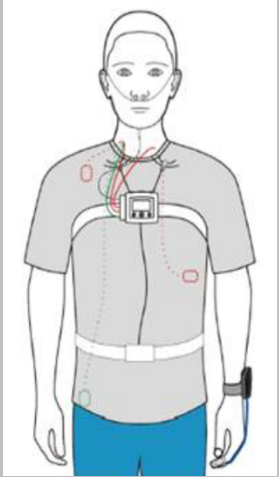
Electrocardiography (ECG)	Two-channel bipolar thoracic ECG
Pulse rate	PPG (pulse oximeter)
Blood oxygen saturation	Oximeter finger (SpO_2)
Nasal pressure	Nasal cannula pressure sensor
Thoracic respiratory effort	Thoracic belt sensor (RIP)
Abdominal respiratory effort	Abdominal belt sensor (RIP)
Snoring events	Snore sensor (nasal pressure)
Movement	Movement sensor
Body position	Body position sensor
Activation	Automatically
Software	Noxturnal Version 6.3.2

ECG, electrocardiogram; PPG, photoplethysmography; SpO_2: peripheral capillary oxygen saturation; RIP, respiratory inductance plethysmography

Supplementary Table S2: Comparison of Apnea-Hypopnea Index (AHI) measured by NOX-T3s polygraphy (PG) in the hospital and at home

	Hospital	Home	Δ	p-value
**AHI (events/h)**	16.4 (11.3-27.2)	14.8 (7.0-27.0)	2.6 (-1.4 – 5.2)	0.150

All values are represented as Median and interquartile range (IQR). PSG: polysomnography, AHI: apnea-hypopnea Index

		**Hospital (n=25)**	
		AHI ≥ 15 (n)	AHI < 15 (n)
**Home (n=25)**	AHI ≥ 15 (n)	12	1
	AHI < 15 (n)	1	11
**Intra-individual NtNV categorical change (%)**		8.0	

NtNV, night-to-night variability

Supplementary Table S3: Coordinates of the ROC-curve of the NOX-T3s in predicting clinically relevant OSA (in hospital and at home)

In hospital				At home			
**AHI cut-off (events/h)**	Sensitivity (%)	Specificity (%)	Youden's J Index	**AHI cut-off (events/h)**	Sensitivity (%)	Specificity (%)	Youden's J Index
**1,40**	100,0	0,0	0,000	**1,00**	100,0	0,0	0,000
**3,25**	100,0	9,1	0,091	**2,55**	100,0	8,3	0,083
**5,10**	100,0	18,2	0,182	**3,65**	100,0	16,7	0,167
**6,60**	94,4	18,2	0,126	**4,25**	100,0	25,0	0,250
**8,10**	94,4	27,3	0,217	**4,70**	100,0	33,3	0,333
**9,45**	94,4	36,4	0,308	**5,80**	92,9	33,3	0,262
**9,85**	88,9	36,4	0,253	**6,80**	92,9	41,7	0,345
**11,25**	88,9	45,5	0,343	**7,85**	92,9	50,0	0,429
**12,65**	88,9	54,5	0,434	**9,00**	92,9	58,3	0,512
**13,05**	83,3	54,5	0,379	**9,65**	92,9	66,7	0,595
**13,45**	83,3	63,6	0,470	**11,10**	**92,9**	**75,0**	**0,679**
**14,00**	77,8	63,6	0,414	**13,30**	85,7	75,0	0,607
**14,55**	77,8	72,7	0,505	**14,40**	78,6	75,0	0,536
**15,35**	**77,8**	**81,8**	**0,596**	**14,75**	71,4	75,0	0,464
**16,25**	72,2	81,8	0,540	**15,20**	64,3	75,0	0,393
**17,00**	66,7	81,8	0,485	**15,60**	57,1	75,0	0,321
**17,70**	61,1	81,8	0,429	**17,65**	50,0	75,0	0,250
**18,25**	61,1	90,9	0,520	**20,50**	50,0	83,3	0,333
**19,40**	50,0	90,9	0,409	**21,80**	42,9	83,3	0,262
**21,00**	44,4	90,9	0,354	**24,10**	35,7	83,3	0,190
**23,55**	44,4	100,0	0,444	**28,05**	35,7	91,7	0,274
**27,15**	38,9	100,0	0,389	**30,60**	28,6	91,7	0,202
**29,20**	33,3	100,0	0,333	**33,60**	21,4	91,7	0,131
**33,40**	27,8	100,0	0,278	**36,05**	14,3	91,7	0,060
**37,70**	22,2	100,0	0,222	**37,15**	14,3	100,0	0,143
**38,00**	16,7	100,0	0,167	**47,45**	7,1	100,0	0,071
**42,35**	11,1	100,0	0,111	**57,70**	0,0	100,0	0,000
**64,35**	5,6	100,0	0,056				
**83,10**	0,0	100,0	0,000				

AHI, apnea-hypopnea index (events/h); OSA, obstructive sleep apnea, ROC, receiver operating characteristic curve

2 Supplementary Figures

Supplementary Figure S1: Bland-Altman analysis: Agreement between the Apnea-Hypopnea Index (AHI) from the polysomnography (PSG) and NOX-T3s polygraphy (PG) during simultaneous in hospital recording (A) and subsequent home recording (B)

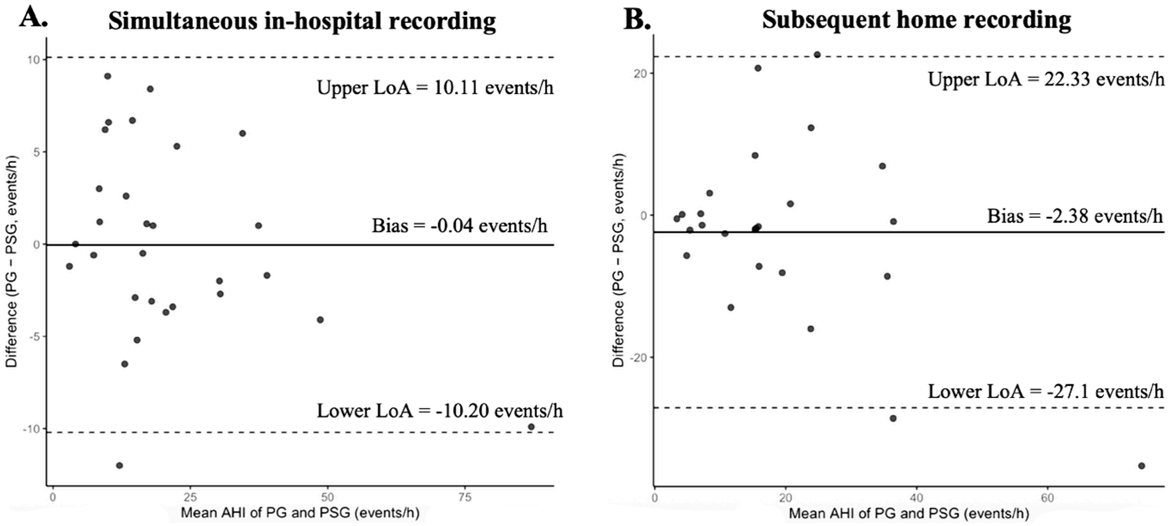

Each dot represents one participant. AHI, apnea-hypopnea index (events/h); PG, cardiorespiratory polygraphy; PSG, polysomnography; LoA, limits of agreement.

3 Supplementary Annexes

Supplementary Annex 1: Comfort PSG Questionnaire

** The first two questions were not applicable when evaluating the polysomnographic examination.*

1. How clear were the instructions for this device?*

0	1	2	3	4	5	6	7	8	9	10

Unclear Very clear

2. Was this device easy to attach?*

0	1	2	3	4	5	6	7	8	9	10

Very difficult Very easy

3. Did you experience any discomfort during your sleep due to this device?

0	1	2	3	4	5	6	7	8	9	10

No discomfort Very uncomfortable

4. How did you sleep last night?

0	1	2	3	4	5	6	7	8	9	10

Restless Peaceful

5. In general, what score would you give based on (the difficulty of attachment and) comfort of this device?

0	1	2	3	4	5	6	7	8	9	10

Bad Good

6. Did you experience any symptoms last night that may indicate atrial fibrillation (palpitations, irregular pulse, shortness of breath, chest pain)?

0	1	2	3	4	5	6	7	8	9	10

No symptoms Severe symptoms

7. Did this device interfere with your sleep?

0	1	2	3	4	5	6	7	8	9	10

No interference Severe interference

8. What score would you give to your sleep last night?

0	1	2	3	4	5	6	7	8	9	10

Poor Good

Other remarks or suggestions:

…

Supplementary Annex 2: Comfort NOX-T3s PG Questionnaire (home)

1. How clear were the instructions for this device?*

0	1	2	3	4	5	6	7	8	9	10

Unclear Very clear

2. Was this device easy to attach?*

0	1	2	3	4	5	6	7	8	9	10

Very difficult Very easy

3. Did you experience any discomfort during your sleep due to this device?

0	1	2	3	4	5	6	7	8	9	10

No discomfort Very uncomfortable

4. What score would you give to your sleep last night?

0	1	2	3	4	5	6	7	8	9	10

Poor Good

Other remarks or suggestions:

…

Supplementary Annex 3: Patient Experience with Heart Rhythm Monitoring Questionnaire

1. How was your experience with the Fitbit smartwatch in combination with the FibriCheck app?

0	1	2	3	4	5	6	7	8	9	10

Poor — Very good

2. How satisfied are you with the monitoring of your atrial fibrillation using the Fitbit smartwatch?

0	1	2	3	4	5	6	7	8	9	10

Not satisfied — Very satisfied

3. Did wearing the Fitbit smartwatch hinder you during your daily activities?

0	1	2	3	4	5	6	7	8	9	10

No discomfort — Very uncomfortable

4. Did wearing the Fitbit smartwatch interfere with your sleep?

0	1	2	3	4	5	6	7	8	9	10

No discomfort — Very uncomfortable

5. For how long would you be willing to wear such a smartwatch in the long term (fill in)
    - … Weeks
    - … Months
    - … Years

Other remarks or suggestions:

…
